# Shenkang protects renal function in diabetic rats by preserving nephrin expression

**DOI:** 10.1186/s12906-023-04078-6

**Published:** 2023-07-17

**Authors:** Zhihui Qu, Biyao Wang, Yingli Jin, Qingfei Xiao, Ying Zhao, Danning Zhao, Liming Yang

**Affiliations:** 1 Department of Nephrology, The First Hospital of Jilin University, No.3302, Jilin Road, Changchun City, Jilin Province P.R. China; 2 Department of Clinical, Educational and Health Psychology, University College London, 26 Bedford Way, London, WC1H 0AP UK; 3 Department of Pharmacology, College of Basic Medical Science, Jilin University, Changchun, 130021 P.R. China; 4 Department of Medical Informatics, School of Public Health, Jilin University, Changchun, 130021 P.R. China

**Keywords:** Diabetic nephropathy, Shenkang injection, Albumin to creatinine ratio, Nephrin, TGF-β1

## Abstract

**Background:**

Shenkang injection has been used clinically to lower creatinine levels. This study explored the mechanism of Shenkang injection on protecting kidney function from hyperglycemia-mediated damage.

**Methods:**

This study utilized a STreptoZotocin (STZ)-induced rat model of diabetes. In total, 60 rats were randomized into either the control group (n = 15) injected with vehicle or treatment group (n = 45) injected with STZ to induce hyperglycemia. Eight weeks after diabetes onset, diabetic rats were further randomized to receive different treatments for 4 consecutive weeks, including vehicle (diabetic nephropathy group, n = 15), Shenkang (n = 15), or Valsartan (n = 15). At 12 weeks, a series of urine and blood measures were examined and damage to the kidney tissue was examined using histology. Expression of nephrin and transforming growth factor-β1 (TGF-β1) were characterized using immunohistochemistry and Western blot.

**Results:**

Compared to the control group, rats in the diabetic nephropathy group showed significant kidney damage demonstrated by high kidneyindex, high levels of urinary albumin, albumin/creatinine ratio (ACR), blood urea nitrogen as well as histological evidence. Shenkang injection significantly improved kidney function in the diabetic rats by decreasing kidney index, ACR, and serum creatinine. Shenkang treatment also mitigated kidney damage, improved nephrin expression, and decreased TGF-β1 expression in the kidneys.

**Conclusions:**

Shenkang treatment protected renal function in diabetic rats by increasing nephrin expression, which protects diabetic rats from hyperglycemia-mediated kidney damage.

**Supplementary Information:**

The online version contains supplementary material available at 10.1186/s12906-023-04078-6.

## Background

According to the International Diabetes Federation, the incidence of diabetes is expected to be at least 783.2 million by 2045 [1]. About 30–40% of patients with diabetes develop diabetic nephropathy, one of the most common complications of diabetes, leading to chronic kidney disease (CKD) and renal failure [2]. Diabetic nephropathy is a kind of diabetic microangiopathy characterized by glomerular damage caused by long-term hyperglycemia, hypertension, microcirculatory disorders, and hypercoagulability [3, 4]. The current management of diabetic nephropathy focuses on tight glycemic control and antihypertensive/lipid-lowering therapies; however, these interventions are limited in the progression of diabetic nephropathy in a large proportion of patients [5, 6]. Many CKD patients rely on kidney dialysis or transplantation to improve kidney function [7]. Thus, diabetes is imposing a growing economic burden in patients in China. Diabetes-related complications and comorbidities have a great impact on medical financial costs [8]. Therefore, discovery of new safe therapeutic drugs is of high significance for the management of patients with diabetic nephropathy.

During the pathogenic process of diabetic nephropathy, hyperglycemia stimulates abnormal cell cycling and increases matrix production and matrix protein glycation, leading to renal hypertrophy and increasing glomerular basement membrane thickness [9]. Intra-glomerular hypertension-related glomerular sclerosis, as well as damage to the glomerular podocytes and basement membrane integrity result in proteinuria [10]. Previous studies have shown that transforming growth of factor-β1 (TGF-β1) reduces nephrin expression in the glomeruli and enhances the permeability of the glomerular basement membrane, contributing to the pathogenesis of diabetic nephropathy [11–13]. Conceivably, therapeutic strategies to enhance nephrin expression may be valuable for supporting the survival of podocytes and preserving the integrity of the glomerular basement membrane, inhibiting the progression of diabetic nephropathy.

Shenkang is an injectable medicine containing mixed extracts from four traditional Chinese medicines, including rhubarb (*Rheum officinale Baill*), astragalus (*Astragalus membranaceus Bunge*), salvia miltiorrhiza (*Salvia miltiorrhiza Bunge*), and safflower (*Carthamus tinctorius L.*) [14]. Chemically, Shenkang contains emodin, rhein, danshensu, and salvianolic acid A, which are considered the main bioactive compounds [15, 16]. The combination of these traditional Chinese medicines may partly play a role in the antifibrotic mechanism by inhibiting the TGF-β/Smad3 pathway [17]. Previous studies have shown that Shenkang benefits patients with CKD by reducing proteinuria and serum creatinine [18–20]. Its major active component emodin can inhibit the proliferation and induce apoptosis of mesangial cells in high glucose conditions [21]. Shenkang has also been shown to decrease TGF-β1 expression, as well as reduce tubulointerstitial pathological changes and the glomerular matrix contents in animal models of diabetic nephropathy [21–23]. However, there is limited literature on the treatment effects of Shenkang on regulating nephrin expression and the integrity of the glomerular basement membrane during the process of diabetic nephropathy.

In this study, we employed a rat model of diabetic nephropathy to examine the treatment effect of Shenkang on kidney function and its potential role in regulating nephrin expression and glomerular basement membrane integrity.

## Methods

### Animals

Six- to seven-week old male Wistar rats (220 ± 20 g) were obtained from the Animal Laboratory of Jilin University, Changchun, China and housed individually in a specific pathogen-free facility with a cycle of 12:12-h light/dark and free access to standard rat chow and water. The experiments were performed according to the Guidelines for the Care and Use of Laboratory Animals, and the experimental protocol was approved by the Animal Research and Care of Committee, Jilin University.

### Animal model and treatments

**Fig. 1:**
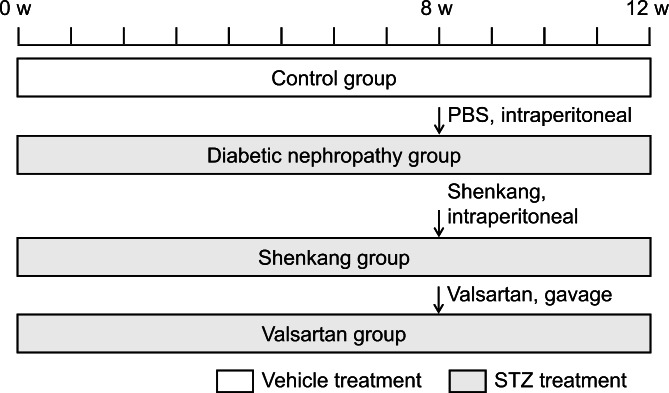
Flow chart of animal experiments

After being acclimatized in the experimental environment for one week, 60 male Wistar rats were grouped randomly according to the random number table method. Fifteen rats were injected intraperitoneally with vehicle (0.1 mmol/L citrate buffer, pH 4.2,) as the control group, and 45 rats were injected intraperitoneally with 65 mg/kg streptozotocin (STZ; Sigma, USA) to induce hyperglycemia. Blood glucose levels were measured using the OneTouch blood glucose meter (Lifescan, China). When the blood glucose concentration was ≥ 16.7 mmol/L for two consecutive days, the rats were diagnosed with diabetes. Eight weeks after diabetes onset, the rats were randomized to receive one of the following treatments: (1) vehicle PBS (no treatment) by intraperitoneal injection as the diabetes nephropathy group (n = 15); (2) Shenkang treatment (2.6 ml/kg ShiJi Shengkang Pharmaceutical Industry Co. Ltd. China) by intraperitoneal injection as the Shenkang group (n = 15) [24]; or (3) Valsartan treatment (15.5 mg/kg Valsartan Beijing Novartis Pharma, China) by gavage as the Valsartan group (n = 15). All treatments were performed daily for four weeks. The animal experimental procedure is summarized in Fig. 1.

At the end of the experiment (12 weeks after diabetes onset), body weights of individual rats were measured. The 24-h urine samples were collected from individual rats in metabolic cages (Kangway Medical Sciences, China). After centrifugation, the urine samples were used to measure levels of urinary protein, albumin, creatinine, and urea nitrogen. In addition, abdominal aortic blood was collected from individual rats and their serum samples were prepared for measuring the levels of serum cholesterol, triglyceride, low density lipoprotein, urea nitrogen, and creatinine. Subsequently, the rats were anaesthetized by inhalation of 4% isoflurane and sacrificed by cervical dislocation, and their kidneys were dissected and wet-weighed to calculate the kidney index (the ratios of kidney weight to body weight). One part of the kidney samples was fixed in 10% formalin overnight and paraffin-embedded. The remaining kidney samples were immediately frozen in liquid nitrogen and stored at -80 °C.

### Laboratory tests

The levels of serum cholesterol, triglyceride, low density lipoprotein, urea nitrogen and creatinine, urinary protein, albumin, nitrogen, and creatinine in individual rats were measured using an auto-biochemical analyzer (Hitachi QA36, Hitachi, Japan) in the First Hospital of Jilin University.

### Pathological examination

The paraffin-embedded kidney tissue Sect. (4 μm) were dewaxed, rehydrated, and stained with hematoxylin and eosin (H&E). The other kidney tissue sections were stained with periodic acid-Schiff’s (PAS), as described previously [25]. A total of 20 glomeruli from the individual rats were captured and imaged under a light microscope (OlympusBX51, Japan). The cross-sectional areas of individual glomeruli were measured using the HPIAS-1000 color image analysis system.

### Transmission electron microscope (TEM)

Renal cortex specimens (1 mm^3^) from individual kidney tissues were fixed with 4% glutaraldehyde (Sigma) for 2 h, and 1% osmic acid (Beijing Cowen Biotech, China) for 1 h. After dehydration, the samples were embedded in Epon epoxy resin 812. Ultrathin Sect. (80 nm) were stained with uranyl acetate and lead citrate (Sigma). The sections were photographed under a TEM (JEM-1010, Jeol Electron, Japan).

### Immunohistochemistry

The impact of Shenkang on nephrin and TGF-β1 protein expression in the glomeruli of rats was determined using immunohistochemistry, as previous described [26]. Briefly, the kidney tissue Sect. (4 µm) were dewaxed, rehydrated, and treated with 3% H_2_O_2_ in methanol, followed by antigen retrieval in citrate buffer (pH 6.0) in a pressure cooker for 10 min. After being blocked with 3% bovine serum albumin, the tissue sections were incubated with goat anti-nephrin or anti-TGF-β1 (1:100, Sigma) overnight at 4°C. Normal goat sera (10%) served as the negative control. After being washed, the bound antibodies were reacted with biotinylated rabbit anti-goat IgG (Mainxin Biotech, China). Subsequently, the sections were incubated with peroxidase (HRP)-conjugated streptavidin (Sigma) and visualized using 3,3’-diaminobenzidine (DAB, Sigma). The kidney glomeruli were imaged and photographed under a light microscope.

### Western blot analysis

Western blot analysis was performed as previous described [20]. The collected renal cortex tissues were homogenized in lysis buffer containing protease inhibitors and centrifuged. The protein concentrations were determined using the bicinchoninic acid (BCA) protein assay kit, according to the manufacturer’s instructions (ThermoFisher Scientific, USA). Individual lysates (50 µg/lane) were separated by sodium dodecyl sulfate polyacrylamide gel electrophoresis (SDS-PAGE) on 10% gels and transferred onto PVDF (polyvinylidene fluoride) membranes (Dingguo). After being blocked with 5% fat-free dry milk in TBS-T buffer for 1–2 h at room temperature, the membranes were incubated with goat anti-nephrin, anti-TGF-β1 or anti-GAPDH antibodies, respectively. The bound antibodies were reacted with HRP-conjugated rabbit anti-goat IgG antibodies and visualized using the enhanced chemiluminescent reagent (ThermoFisher Scientific). The relative expression of target protein to the control GAPDH was determined using densitometric scanning with the Quantity One software (Bio-Rad, USA). The results are demonstrated as the ratios of target protein to the internal reference.

### Statistical analysis

Body, urine, and blood measures in different groups of rats are descriptively reported as mean and standard deviation (SD). Normality in data distribution was examined using the Shapiro-Wilk test, with a *P*-value > 0.05 suggesting normal distribution. Differences among groups were tested using analysis of variance (ANOVA) given normally distributed data and Kruskal-Wallis test by ranks for non-normally distribute data. A *P*-value < 0.05 was considered statistically significant. Post hoc comparisons were performed using the Tukey HSD test given significant effects in ANOVA and using the Pairwise Wilcoxon Rank Sum test given significant effects in the Kruskal-Wallis test. Effect sizes were calculated to quantify the size of the difference between two groups, η2 and ω2 (corrected for small sample size) for ANOVA [27], η2 for Kruskal–Wallis test by ranks [28]. The interpretations of effect sizes, namely η2 and ω2, were: 0.01 represents a small effect size, 0.06 represents a medium effect size, and 0.14 represents a large effect size [6]. Analyses were conducted in R version 3.6.1 [29].

## Results

### Shenkang ameliorates kidney function in diabetic rats

Descriptive statistics for body, urine, and blood measures in the different groups of diabetic rats (diabetic nephropathy, Shenkang, Valsartan), as well as the control group of healthy rats were reported in Table 1. Prior to examination of the differences between groups, Shapiro-Wilk tests were performed to test the assumption of normal distribution (Table 1, Supplemental Table 1). ANOVA was conducted to compare group differences in kidney weight/body weight, low-density lipoprotein, and serum creatinine, given the data followed a normal distribution (pKW/BW = 0.53, pLDL = 0.16, pserum creatinine = 0.68). Then, Tukey HSD tests were used for Post hoc pairwise comparisons. Examination of differences between groups was performed using the Kruskal-Wallis Tests given the data were not normally distributed (*P* < 0.05) followed by Pairwise Wilcoxon Rank Sum test (Table 1, Supplemental Table 2).

**Table 1:** Descriptive statistics of diabetic rats in randomized groups

	Control				Diabetic Nephropathy				Shenkang				Valsartan				Group difference	
	N	Mean	SD		N	Mean	SD		N	Mean	SD		N	Mean	SD		*P*value	Pairwise difference
Body Measure																		
KW/BW (%)	15	0.41	0.12		15	0.67	0.12		15	0.52	0.08		15	0.54	0.14		< 0.01	NC-DN, NC-V, DN-SK, DN-V
Urine measures																		
Urinary protein (mg/L)	15	647.67	375.52		15	852.27	585.58		15	710.6	392.24		15	1103	582.72		0.08	
Urinary albumin (mg/L)	15	15.03	4.77		15	24.78	9.83		15	19.13	6.67		15	15.03	6.03		< 0.01	NC-DN, DN-V
Urine urea nitrogen (mmol/L)	15	436.27	212.65		15	457.17	212.4		15	435.4	213.89		15	389.5	108.89		0.96	
Urine creatinine (µmol/L)	15	4327.4	1915.04		15	3367.3	1290.13		15	3765.2	1057.02		15	2747.35	887.8		0.02	NC-V
ACR (ug/mg)	15	33.48	11.86		15	68.51	26.28		15	45.62	12.47		15	50.18	15.69		< 0.01	NC-DN, NC-V, DN-SK, DN-V
Blood measures																		
CHO (mmol/L)	15	1.62	0.42		15	1.87	0.42		15	1.83	0.39		15	1.53	0.23		0.02	DN-V
TG (mmol/L)	15	0.58	0.22		15	0.82	0.34		15	0.99	0.25		15	0.9	0.61		< 0.01	NC-SK
LDL (mmol/L)	15	0.41	0.1		15	0.54	0.17		15	0.57	0.16		15	0.45	0.13		0.01	NC-SK
BUN (mmol/L)	15	10.19	3.4		15	18.47	6.13		15	14.11	4.45		15	18.53	6.84		< 0.01	NC-DN, NC-SK, NC-V
Serum creatinine (µmol/L)	15	135.4	11.79		15	143.67	24.84		15	111.87	11.51		15	137.47	17.49		< 0.01	NC-SK, DN-SK, V-SK

Notes. KW/BW = kidney weight/body weight, ACR = albumin/creatinine ratio, CHO = Total cholesterol, TG = triglyceride, LDL=

Low-density lipoprotein, BUN = blood urea nitrogen, SD = standard deviation. Differences among groups were tested using analysis of variance (ANOVA) given the data is normally distributed (KW/BW, LDL) and using Kruskal–Wallis test by ranks given the data is not normally distributed (urinary protein, urinary albumin, urine urea nitrogen, urine creatinine, ACR, CHO, TG). Post hoc pairwise comparisons were conducted by Tukey HSD test given the data is normally distributed (KW/BW, LDL) and Pairwise Wilcoxon Rank Sum test given the data is not normally distributed (urinary protein, urinary albumin, urine urea nitrogen, urine creatinine, ACR, CHO, TG).

There were significant differences in kidney index (KW/BW) among the groups (F(3,56) = 12.28, *P* < 0.01, η2 = 0.4, ω2 = 0.36). Mean ratios of kidney body weight in the diabetic nephropathy group were significantly higher than that in the control group (*P* < 0.01). Compared with rats in the diabetic nephropathy group, the rats that received Shenkang (*P* = 0.01) and Valsartan (*P* = 0.02) had significantly reduced ratios of kidney to body weight. The kidney index in the Shenkang group was similar to that of control group (*P* = 0.06).

For urine measurements, there were significant differences in urinary albumin (x2 = 15.08, df = 3, *P* < 0.01, η2 = 0.22), urine creatinine (x2 = 10.04, df = 3, *P* = 0.02, η2 = 0.13), and albumin/creatinine ratio (ACR) (x2 = 23.43, df = 3, *P* < 0.01, η2 = 0.36). Compared to the rats in the control group, those in the diabetic nephropathy group had significantly higher levels of urinary albumin (*P* < 0.01) and ACR (*P* < 0.01). The Shenkang (*P* < 0.01) and Valsartan (*P* = 0.03) groups showed significantly lower ACR compared with the diabetic nephropathy group. For urinary albumin, despite the Valsartan group showing a significantly lower level compared with the diabetic nephropathy group (*P* < 0.01), both the Shenkang (*P* = 0.12) and Valsartan (*P* = 0.93) groups showed similar levels compared with the control group. The urine creatinine level was lower in the Valsartan group compared to the control group (*P* = 0.02) and Shenkang group (*P* = 0.04). No significant difference was found among the four groups for urinary protein (x2 = 6.85, df = 3, *P* = 0.08, η2 = 0.07) or urine urea nitrogen (x2 = 0.31, df = 3, *P* = 0.96, η2=-0.05).

Significant group differences were also found in all blood measures, including total cholesterol (x2 = 9.42, df = 3, *P* = 0.02, η2 = 0.11), triglyceride (x2 = 13.92, df = 3, *P* < 0.01, η2 = 0.19), low-density lipoprotein (F(3,56) = 4.14, *P* = 0.01, η2 = 0.18, ω2 = 0.14), blood urea nitrogen (x2 = 20.45, df = 3, *P* < 0.01, η2 = 0.31), and serum creatinine (F(3,56) = 9.76, *P* < 0.01, η2 = 0.34, ω2 = 0.30). Rats in the diabetic nephropathy group only showed significantly higher levels of blood urea nitrogen compared with the control group (*P* < 0.01). The mean level of blood urea nitrogen in the Shenkang group was lower compared with the diabetic nephropathy group, but the difference was not statistically significant (*P* = 0.09). All three blood lipid measures, including cholesterol (*P* = 0.53), triglyceride (*P* = 0.23), and low-density lipoprotein (*P* = 0.91), were similar between the Shenkang and diabetic nephropathy groups. The Valsartan group had significantly lower levels of cholesterol compared to the diabetic nephropathy group. It is worth noting that the rats that received Shenkang treatment had significantly reduced serum creatinine levels compared to all other groups (*P* < 0.01).

### Shenkang mitigates kidney damage in diabetic rats

**Fig. 2:**
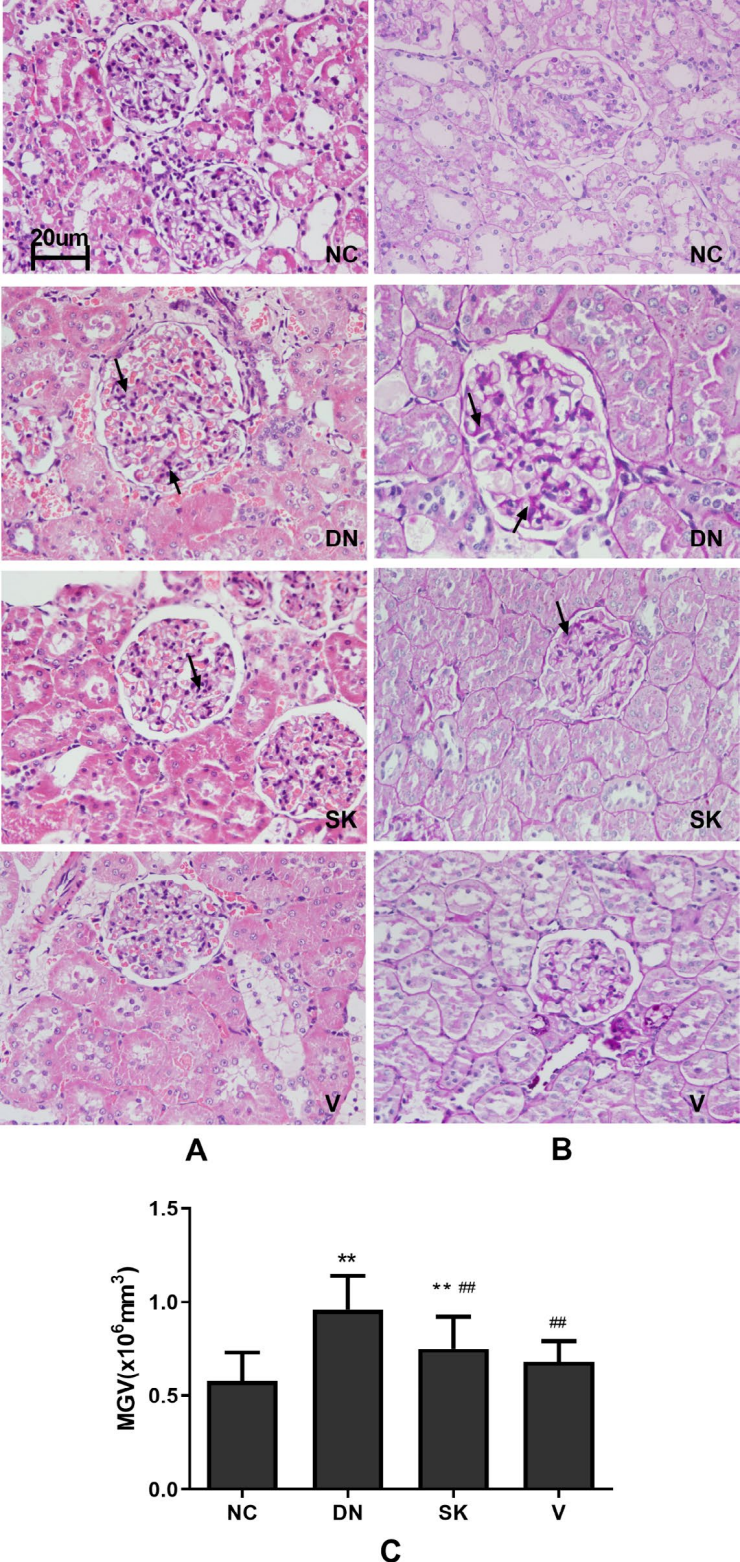
Histological examination of kidney tissue damage in diabetic rats After 4 weeks of treatment with Shenkang or Valsartan, kidney tissue sections from the different groups of rats were stained with H&E and PAS, respectively. The glomerular volumes in individual rats were calculated. Data are representative images (magnification x 400) or expressed as the mean ± SD of each group of rats from three separate experiments. (**A**) H&E and PAS staining; (**B**) Quantitative analysis of the glomerular volumes. Mean glomerular cross-sectional areas (MGA). All groups n = 15. **P < 0.01 vs. the NC group; ##*P* < 0.01 vs. the DN group

Next, we examined the effect of Shenkang treatment on hyperglycemia-induced kidney tissue damage in the different groups of rats. There was an increase in the kidney glomerular areas with proliferative mesangial cells in the diabetic nephropathy group compared with the control group (Fig. 2A-B). Similarly, PAS staining revealed notably occluded blood vessels surrounding the glomeruli, a widened matrix, and filtration membrane and Bowman’s capsule adhesion in the kidney of the diabetic nephropathy group. Such observations demonstrated that long-term hyperglycemia caused kidney damage in the diabetic nephropathy group. Treatment with either Shenkang or Valsartan obviously reduced the pathological changes and mitigated the hyperglycemia-mediated kidney damage in the diabetic rats (Fig. 2A-B). Semi-quantitative analysis revealed that the glomerular volumes in the diabetic nephropathy group of rats were significantly larger than that of the control group (*P* < 0.01, Fig. 2C) while the glomerular volumes in the Shenkang and Valsartan groups were significantly smaller than that in the diabetic nephropathy group of rats (*P* < 0.01 for both).

**Fig. 3:**
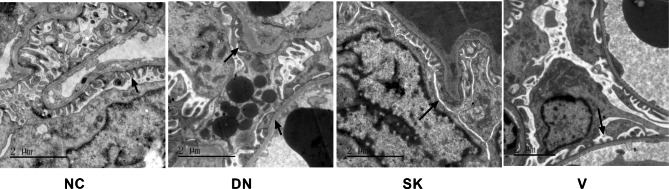
TEM analysis of kidney tissues in the different groups of rats Ultrathin kidney tissue sections from individual groups of rats were stained with uranyl acetate and lead citrate. The sections were photographed under using TEM. Data are representative images (magnification x 10,000) from individual groups of rats. All groups n = 15

TEM analysis showed that the integrity of glomerular basement membrane and the normal structure of the foot process were maintained in the kidney glomeruli of the control group, while the basement membrane was irregular and thickened and there were extensively fused epithelial cell foot processes in the kidney glomeruli of the diabetic nephropathy groups (Fig. 3). In contrast, the pathological changes were notably reduced in the kidney glomeruli of the Shenkang and Valsartan groups. Collectively, these observations indicate that treatment with Shenkang significantly mitigated the hyperglycemia-mediated kidney damage in the diabetic rats.

### Shenkang enhances nephrin expression in the kidneys of diabetic rats

**Fig. 4:**
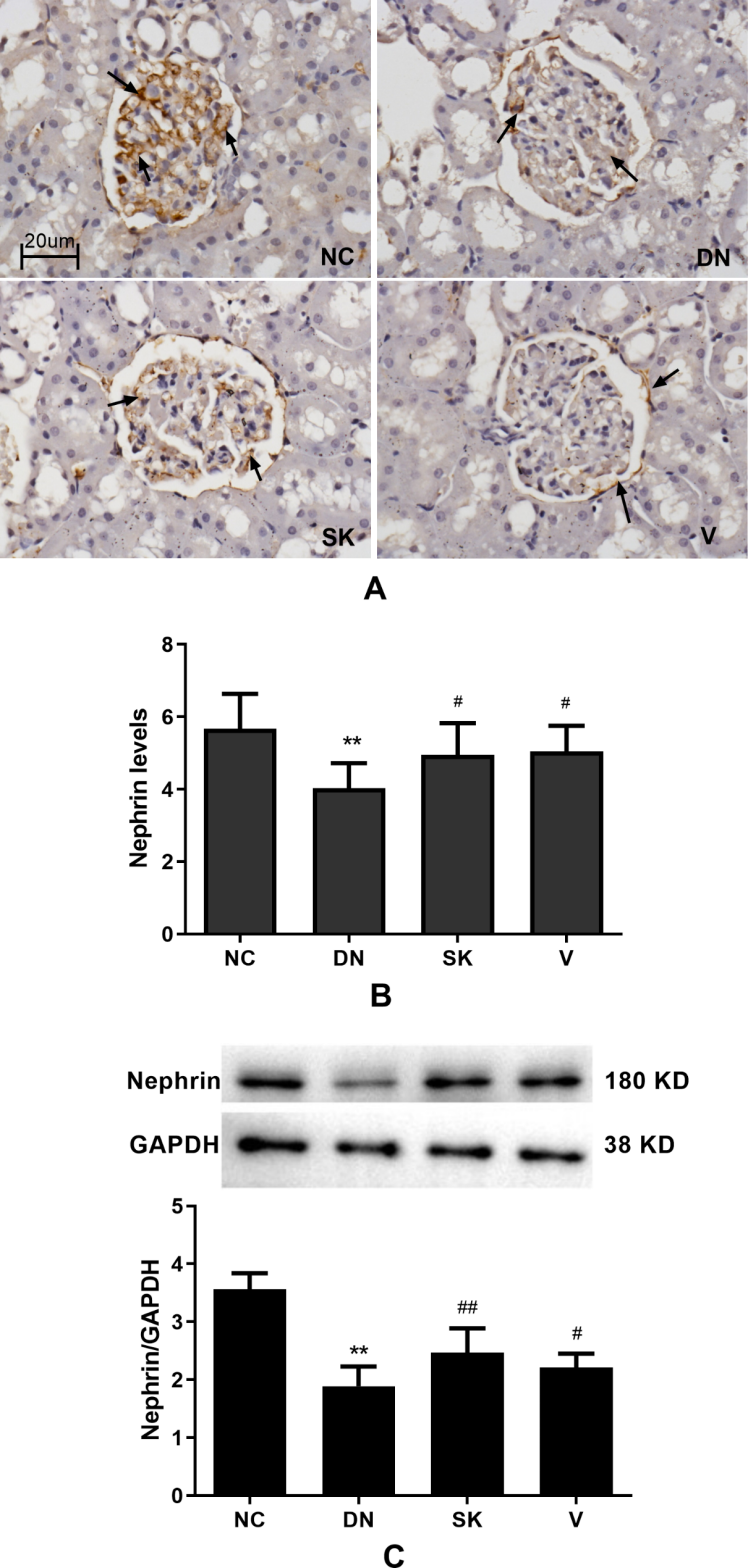
Analysis of nephrin expression in the kidney tissues of different groups of rats Kidney tissue sections were stained with anti-nephrin and HRP-conjugated second antibodies, followed by DAB staining. The signal intensity of nephrin staining in at least 20 glomeruli of each group of rats was measured. The relative nephrin expression in the kidney tissues of individual groups of rats was determined using Western blot analysis. Data are representative images (magnification x 400) or are expressed as the mean ± SD of each group of rats from three separate experiments. (**A**) Immunohistochemistry analysis of nephrin expression; (**B**) Quantitative analysis of the levels of nephrin expression. (**C**) Western blot analysis of the relative levels of nephrin expression. All groups n = 15. **P < 0.01 vs. the NC group; ##*P* < 0.01 vs. the DN group. The full-length blots/gels are presented in Supplementary Fig. 1

To understand the mechanism by which Shenkang improves the kidney function in diabetic rats, the relative expression of nephrin, a necessary protein for the proper function of the filtration barrier [30, 31], was determined using immunohistochemistry and Western blot analyses. As shown in Fig. 3, higher expression of nephrin was observed in the glomerular capillary loop in the kidney tissues of the control group. A similar pattern of slightly weaker nephrin staining was detected in the Shenkang group (Fig. 4A). In contrast, notably lower expression of nephrin was detected in the partial glomerular capillary loop in the diabetic nephropathy and Valsartan groups (Fig. 4A). Quantitative analysis revealed that the nephrin staining in the kidney of the diabetic nephropathy and Valsartan groups was significantly lower than in the Shenkang group (*P* < 0.01), which was similar to that of the control group (Fig. 4B). Further Western blot analysis indicated that the relative expression of nephrin in the kidney tissues of the Shenkang group were lower than that in the control group, but significantly higher than that in the diabetic nephropathy and Valsartan groups (*P* < 0.05 for both, Fig. 4C). Thus, Shenkang treatment significantly enhanced nephrin expression in the kidney of diabetic rats.

### Shenkang mitigates the hyperglycemia-induced TGF-β1 expression in the kidneys of diabetic rats

**Fig. 5:**
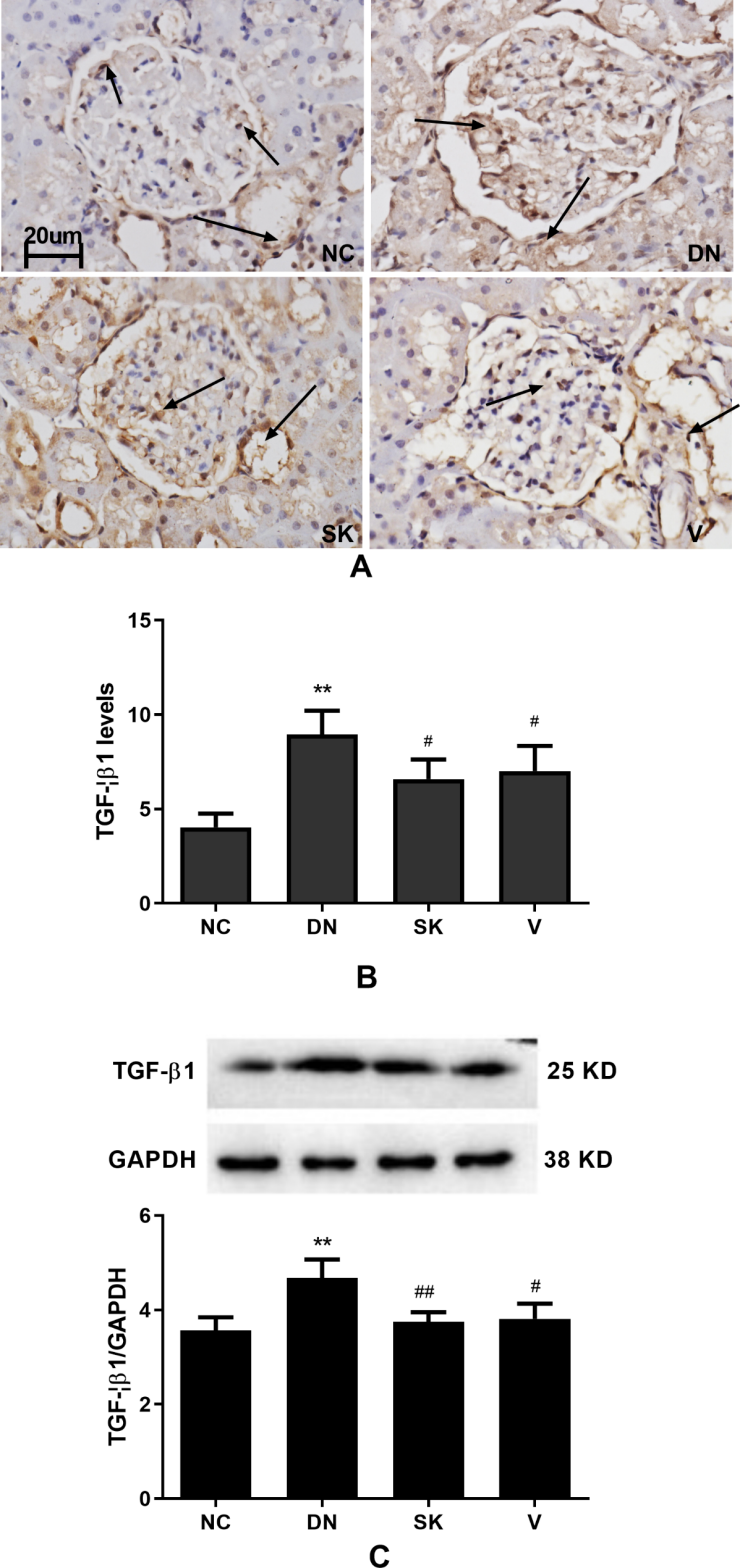
Analysis of TGF-β1 expression in the kidney tissues of different groups of rats Kidney tissue sections were stained with anti-TGF-β1 and HRP-conjugated second antibodies, followed by DAB staining. The signal intensity of TGF-β1 staining in at least 20 glomeruli of each group of rats was measured. The relative TGF-β1 expression in the kidney tissues of individual groups of rats was determined using Western blot analysis. Data are representative images (magnification × 400) or are expressed as the mean ± SD of each group of rats from three separate experiments. (**A**) Immunohistochemistry analysis of TGF-β1 expression; (**B**) Quantitative analysis of the levels of TGF-β1 expression. (**C**) Western blot analysis of the relative levels of TGF-β1 expression. All groups n = 15. **P < 0.01 vs. the NC group; ##*P* < 0.01 vs. the DN group. The full-length blots/gels are presented in Supplementary Fig. 1

TGF-β1 is a pathogenic factor in diabetic nephropathy, as it can promote cell hypertrophy and extracellular matrix accumulation in the mesangium, increasing glomerular permeability [22, 23]. To understand the molecular mechanisms underlying the pharmacologic action of Shenkang, TGF-β1 expression in the kidneys of different groups was determined using immunohistochemistry and Western blot analyses. As shown in Fig. 5A, TGF-β1 expression was mainly located in the cytoplasm of epithelial cells in the glomerular mesangium area. TGF-β1 staining in the kidney tissues of the diabetic nephropathy group was much stronger than in the control, Shenkang, and Valsartan groups. There was no obvious difference in the levels of TGF-β1 expression among the control, Shenkang, and Valsartan groups. Semi-quantitative analysis revealed that the TGF-β1 staining in the diabetic nephropathy group was significantly higher than in the control, Shenkang, and Valsartan groups (*P* < 0.01, *P* < 0.05, Fig. 5B). The TGF-β1 staining in the Shenkang and Valsartan groups remained significantly higher than in the control group (*P* < 0.05). A similar pattern for the relative TGF-β1 expression was detected among the different groups using Western blot analysis (Fig. 5C). Therefore, Shenkang significantly mitigated the hyperglycemia-stimulated TGF-β1 expression in the kidney tissues of the diabetic rats.

## Discussion

CKD is one of the most common conditions that significantly increases the risk of many serious diseases. Epidemiological investigations have shown that CKD has become a serious global health problem. Shenkang injection has been considered an effective treatment for CKD. Our study indicated that Shenkang improved kidney function in a model of diabetic nephropathy. Shenkang treatment reduced the level of hyperglycemia-stimulated TGF-β1 expression and increased nephrin expression in the kidney tissues of diabetic rats. These findings provide new insights into the pharmacological action of Shenkang in the treatment of diabetic nephropathy.

In 2015, Shenkang injection was shown to markedly reduce levels of Scr and BUN, alleviate expression of fibrosis-associated signaling molecules, and reduce expression of TGF-β and phosphorylated Smad3. Meanwhile, in HK-2 cells, exposure to TGF-β and H_2_O_2_ significantly increased the protein expression of renal fibrosis, and generation of oxidative stress was also elevated. Shenkang injection has also been shown to reduce he severity of fibrosis and oxidative damage [18]. Shenkang has been tested for its ability to inhibit cell cycle progression and induce apoptosis of rat renal mesangial cells. It was demonstrated that Shenkang injection and its major active component emodin reduced high glucose‑induced proliferation of renal mesangial cells via inducing cell cycle arrest at G1 phase, as well as reduce cellular apoptosis via upregulating activation of pro‑apoptotic mediators bax and caspase [19]. Shenkang treatment attenuated hyperglycemia-induced increased glomerular volume, as well as reduced mesangial cell proliferation, blood vessel occlusion, and matrix accumulation in the kidneys of diabetic rats [21, 23].

Our results demonstrated that nephrin expression was enhanced and the activity of TGF-β was inhibited by Shenkang treatment under diabetes condition. This finding is consistent with the previous reports showing that TGF-β suppresses nephrin expression [32, 33]. We found that Shenkang treatment significantly prevented hyperglycemia-stimulated TGF-β1 expression in the kidney tissues, similar to that in previous studies [11, 13]. More importantly, Shenkang treatment improved the hyperglycemia-reduced nephrin expression in the glomeruli of diabetic rats. The upregulated nephrin expression reflects the preservation of podocytes from TGF-β1-mediated cell injury and apoptosis [11, 13]. Shenkang treatment preserved the integrity of the glomerular basement membrane and improved kidney function in diabetic rats. These findings explain the mechanism by which Shenkang ameliorates kidney dysfunction in diabetic rats. Therefore, our findings provide new insights into the pharmacological mechanisms of how Shenkang improves kidney function in diabetic rats.

Hyperglycemia can cause chronic inflammation and oxidative stress in the glomeruli during diabetic nephropathy [3, 7]. These conditions, together with blood vessel occlusion, promote glomerular injury and increase glomerular permeability. In traditional Chinese medicines, the components of rhubarb (*Rheum officinale Baill*), astragalus (*Astragalus membranaceus Bunge*), salvia miltiorrhiza (*Salvia miltiorrhiza Bunge*), and safflower (*Carthamus tinctorius L.*) in Shenkang can improve blood circulation and systemic blood rheology, as well as reduce blood viscosity in the glomeruli of diabetic rats. Jiang found that danshensu, an active ingredient, acted as an anti-oxidant, conferring heart protection, renal protection, anti-inflammation, and antithrombosis properties. Danshensu was present at the highest concentration in Shenkang in vivo based on the analytical ultracentrifugation (AUC) data [17]. Previous studies have also shown that these traditional Chinese medicines can improve lipid metabolism and renal blood flow, inhibit platelet aggregation, promote fibrinolysis, and have antioxidant activity [19, 20, 22]. The increased blood flow may limit inflammatory and profibrotic cytokine production, such as TGF-β1, which may in turn protect podocytes from hypoxia and inflammatory cytokine-mediated injury and apoptosis in the glomeruli of diabetic rats. Further investigation of the molecular mechanisms by which Shenkang regulates the expression of TGF-β1 and nephrin in the glomeruli of diabetic animals is needed.

## Conclusions

Our study demonstrated protective effects of Shenkang on mitigating hyperglycemia-mediated kidney damage and ameliorating kidney dysfunction in diabetic rats. Shenkang treatment reduced the level of hyperglycemia-stimulated TGF-β1 expression and increased nephrin expression in the kidney tissues of diabetic rats. Our findings suggest that Shenkang improves kidney function in diabetic rats by maintaining the integrity of the glomerular basement membrane. These findings provide new insights into the pharmacological mechanism by which Shenkang ameliorates kidney dysfunction under diabetic conditions. In a follow-up study, the mechanism by which Shenkang regulates hyperglycemia-stimulated TGF-β1 expression and the relationship between the nephrin and TGF-β1 expression will be investigated in vitro or in vivo.

## Electronic supplementary material

Below is the link to the electronic supplementary material.

Additional file 1: **Supplementary fig. 1**

Additional file 2: **Supplemental table 1**. Tests of group differences between diabetic rats in randomized groups. **Supplemental table 2**. Pairwise group comparison of diabetic rats in randomized groups

## Data Availability

The datasets used and/or analyzed during the current study are available from the corresponding author on reasonable request.

## List of abbreviations

ACR: Albumin/creatinine ratio
ANOVA: Analysis of variance
BUN: Blood urea nitrogen
CHO: Total cholesterol
CKD: Chronic Kidney Disease
ES: Effect size
KW/BW: Kidney weight/body weight
LDL: Low-density lipoprotein
MS: Mean Square
PAS: Periodic acid-Schiff’s
SD: Standard deviation
SS: Sum of Squares
STZ: StreptoZotocin
TEM: Transmission electron microscope
TG: Triglyceride
TGF-β1: Transforming growth factor-β1
