# Supplementary figures and images for: Shenkang protects renal function in diabetic rats by preserving nephrin expression
Source: BMC Complement Med Ther. 2023 Jul 17;23:244. doi: 10.1186/s12906-023-04078-6 (PMC10353195; doi:10.1186/s12906-023-04078-6)

**Supplementary fig. 1**


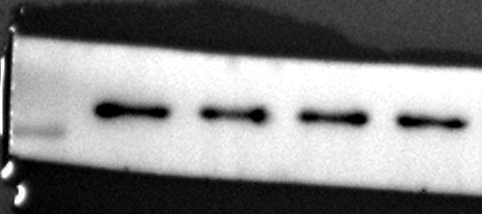
 GAPDH-2(1)


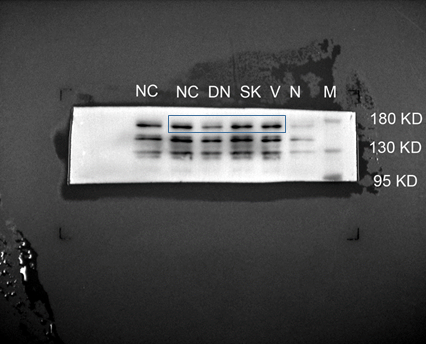
 Nephrin-311


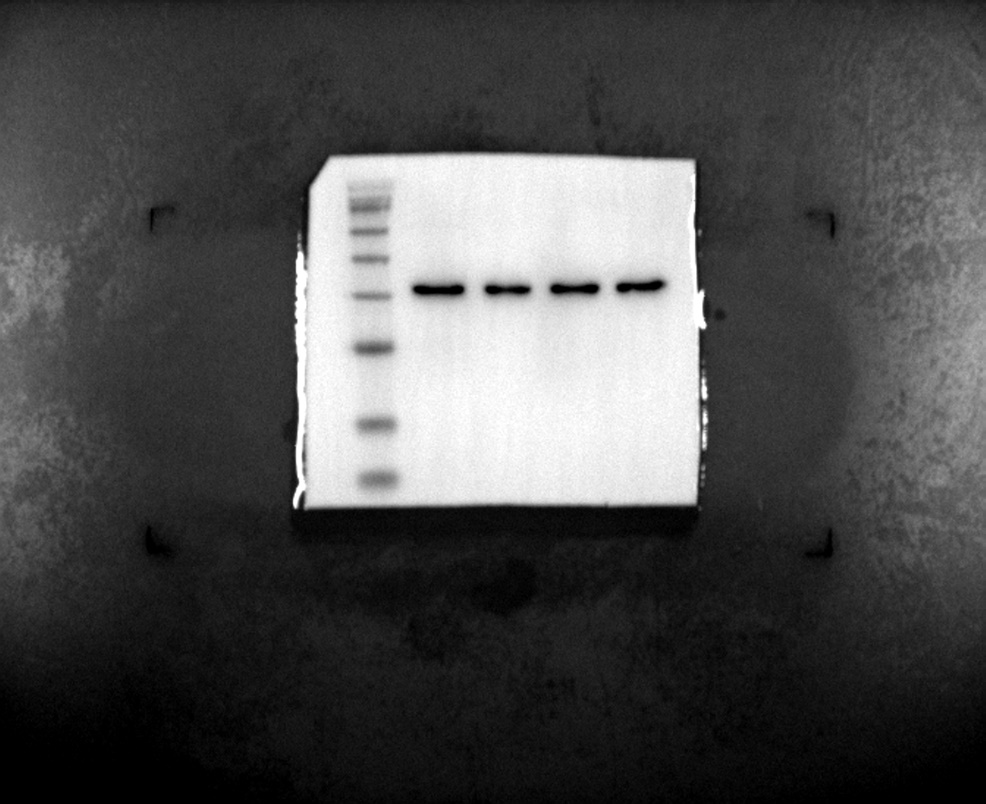
 GAPDH-1(1)


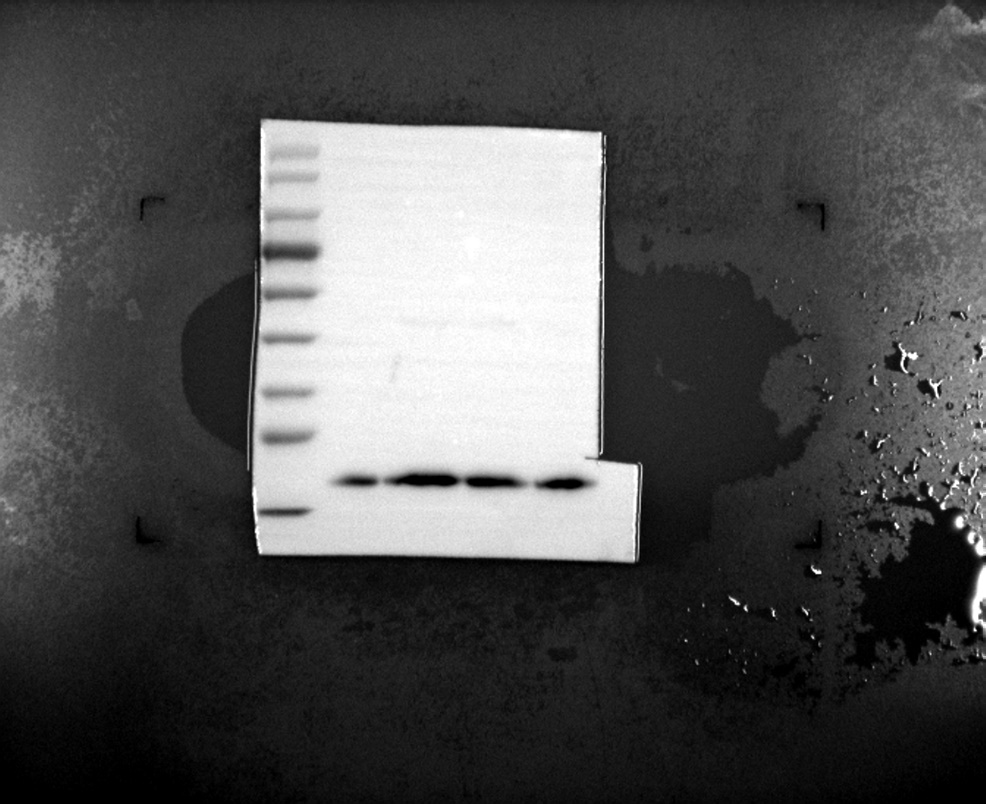
 TGF-1(1)

Supplement: Supplementary file 1 — Additional file 1: Supplementary fig. 1 [file 12906_2023_4078_MOESM1_ESM.docx]
