# Supplementary material for: Shenkang protects renal function in diabetic rats by preserving nephrin expression
Source: BMC Complement Med Ther. 2023 Jul 17;23:244. doi: 10.1186/s12906-023-04078-6 (PMC10353195; doi:10.1186/s12906-023-04078-6)
Supplement: Supplementary file 2 — Additional file 2: Supplemental table 1. Tests of group differences between diabetic rats in randomized groups. Supplemental table 2. Pairwise group comparison of diabetic rats in randomized groups [file 12906_2023_4078_MOESM2_ESM.docx]

**Supplemental table 1.** Tests of group differences between diabetic rats in randomized groups

|  | Distribution test | |  | | | Analysis of variance (ANOVA) | | | | | | |  | | Kruskal–Wallis test by ranks | | | | |
| --- | --- | --- | --- | --- | --- | --- | --- | --- | --- | --- | --- | --- | --- | --- | --- | --- | --- | --- | --- |
|  | *Statistic* | *P* | |  | *SS* | | *df* | *MS* | *F* | *P* | *η^2^* | *𝜔^2^* | |  | *𝜒^2^* | *df* | *P* | *η^2^* |  |
| Body Measure |  |  | |  |  | |  |  |  |  |  |  | |  |  |  |  |  |  |
| KW/BW (%) | 0.98 | 0.53 | |  | 0.51 | | 3 | 0.17 | 12.28 | <0.01 | 0.4 | 0.36 | |  |  |  |  |  |  |
| Urine measures |  |  | |  |  | |  |  |  |  |  |  | |  |  |  |  |  |  |
| Urinary protein (mg/L) | 0.89 | <0.01 | |  |  | |  |  |  |  |  |  | |  | 6.85 | 3 | 0.08 | 0.07 |  |
| Urinary albumin (mg/L) | 0.86 | <0.01 | |  |  | |  |  |  |  |  |  | |  | 15.08 | 3 | <0.01 | 0.22 |  |
| Urine urea nitrogen (mmol/L) | 0.88 | <0.01 | |  |  | |  |  |  |  |  |  | |  | 0.31 | 3 | 0.96 | -0.05 |  |
| Urine creatinine (umol/L) | 0.89 | <0.01 | |  |  | |  |  |  |  |  |  | |  | 10.04 | 3 | 0.02 | 0.13 |  |
| ACR (µg/mg） | 0.87 | <0.01 | |  |  | |  |  |  |  |  |  | |  | 23.43 | 3 | <0.01 | 0.36 |  |
| Blood measures |  |  | |  |  | |  |  |  |  |  |  | |  |  |  |  |  |  |
| CHO (mmol/L) | 0.94 | <0.01 | |  |  | |  |  |  |  |  |  | |  | 9.42 | 3 | 0.02 | 0.11 |  |
| TG (mmol/L) | 0.87 | <0.01 | |  |  | |  |  |  |  |  |  | |  | 13.92 | 3 | <0.01 | 0.19 |  |
| LDL (mmol/L) | 0.97 | 0.16 | |  | 0.26 | | 3 | 0.09 | 4.14 | 0.01 | 0.18 | 0.14 | |  |  |  |  |  |  |
| BUN (mmol/L) | 0.94 | 0.01 | |  |  | |  |  |  |  |  |  | |  | 20.45 | 3 | <0.01 | 0.31 |  |
| Serum creatinine (µmol/L) | 0.99 | 0.68 | |  | 8743 | | 3 | 2914.33 | 9.76 | <0.01 | 0.34 | 0.3 | |  |  |  |  |  |  |

Notes. KW/BW = kidney weight/body weight, ACR = albumin/creatinine ratio, CHO = Total cholesterol, TG = triglyceride, LDL= Low-density lipoprotein, BUN = blood urea nitrogen, ANOVA = analysis of variance, SS = Sum of Squares, MS = Mean Square, ES = effect size. Shapiro-Wilk test p-value > 0.05 suggests normal data distribution. Differences among groups were tested using analysis of variance (ANOVA) given the data is normally distributed and using Kruskal–Wallis test by ranks given the data is not normally distributed.

**Supplemental table 2.** Pairwise group comparison of diabetic rats in randomized groups

|  | Tukey HSD test *P*-value | | | | | |  | Pairwise Wilcoxon Rank Sum test *P*-value | | | | |
| --- | --- | --- | --- | --- | --- | --- | --- | --- | --- | --- | --- | --- |
|  | NC-DN | NC-SK | NC-V | DN-SK | DN-V | V-SK |  | NC-DN | NC-SK | NC-V | DN-SK | DN-V |
| Body Measure |  |  |  |  |  |  |  |  |  |  |  |  |
| KW/BW (%) | <0.01 | 0.06 | 0.02 | 0.01 | 0.02 | 0.96 |  |  |  |  |  |  |
| Urine measures |  |  |  |  |  |  |  |  |  |  |  |  |
| Urinary protein (mg/L) |  |  |  |  |  |  |  | 0.69 | 0.71 | 0.07 | 0.71 | 0.4 |
| Urinary albumin (mg/L) |  |  |  |  |  |  |  | <0.01 | 0.12 | 0.93 | 0.12 | <0.01 |
| Urine urea nitrogen (mmol/L) |  |  |  |  |  |  |  | >.99 | >.99 | >.99 | >.99 | >.99 |
| Urine creatinine (umol/L) |  |  |  |  |  |  |  | 0.23 | 0.57 | 0.02 | 0.39 | 0.37 |
| ACR (ug/mg) |  |  |  |  |  |  |  | <0.01 | 0.03 | 0.01 | <0.01 | 0.03 |
| Blood measures |  |  |  |  |  |  |  |  |  |  |  |  |
| CHO (mmol/L) |  |  |  |  |  |  |  | 0.17 | 0.67 | 0.17 | 0.53 | 0.04 |
| TG (mmol/L) |  |  |  |  |  |  |  | 0.09 | <0.01 | 0.09 | 0.23 | 0.97 |
| LDL (mmol/L) | 0.08 | 0.01 | 0.85 | 0.91 | 0.36 | 0.11 |  |  |  |  |  |  |
| BUN (mmol/L) |  |  |  |  |  |  |  | <0.01 | 0.03 | <0.01 | 0.09 | 0.97 |
| Serum creatinine (umol/L) | 0.56 | <0.01 | 0.99 | <0.01 | 0.76 | <0.01 |  |  |  |  |  |  |

Notes. NC=(negative) Control, DN=Diabetic Nephropathy, SK=Shenkang, V=Valsartan; KW/BW = kidney weight/body weight, ACR = albumin/creatinine ratio, CHO = Total cholesterol, TG = triglyceride, LDL= Low-density lipoprotein, BUN = blood urea nitrogen. Post hoc pairwise comparisons were conducted by Tukey HSD test given the data is normally distributed and Pairwise Wilcoxon Rank Sum test given the data is not normally distributed.
